# Research advances in traditional Chinese medicine formulae and active components targeting lipid metabolism for hepatocellular carcinoma therapy

**DOI:** 10.3389/fphar.2025.1528671

**Published:** 2025-04-25

**Authors:** Yang Liu, Jie Yang, Fenghua Yu, Li Li, Ning Zhao, Cheng Lu, Aiping Lu, Xiaojuan He

**Affiliations:** ^1^ Institute of Basic Research in Clinical Medicine, China Academy of Chinese Medical Sciences, Beijing, China; ^2^ Law Sau Fai Institute for Advancing Translational Medicine in Bone and Joint Diseases, School of Chinese Medicine, Hong Kong Baptist University, Kowloon, Hong Kong SAR, China; ^3^ Institute of Systems Medicine and Health Sciences, School of Chinese Medicine, Hong Kong Baptist University, Kowloon, Hong Kong SAR, China; ^4^ Shanghai GuangHua Hospital of Integrated Traditional Chinese and Western Medicine, Institute of Arthritis Research, Shanghai Academy of Chinese Medical Sciences, Shanghai, China

**Keywords:** hepatocellular carcinoma, lipid metabolism, traditional Chinese medicine, formulae, active components

## Abstract

Hepatocellular carcinoma (HCC) has a relatively poor prognosis and a high degree of malignancy. However, the therapeutic drugs are limited. In recent years, abnormal lipid metabolism and its important role in HCC has been reported, and emerging studies found that some formulae and active components of traditional Chinese medicine (TCM) can regulate abnormal lipid metabolism in HCC, showing their good application prospects. Therefore, this article summarizes the changes and the roles of lipid metabolites in HCC progression, and discusses the role of formulae and active components of TCM for the treatment of HCC based on their regulation on abnormal lipid metabolism. A deeper understanding of their relationship may help the precise use of these formulae and active components in HCC.

## 1 Introduction

Hepatocellular carcinoma (HCC) is a common cancer affecting individuals globally (Vogel et al., 2022). Half of the world’s HCC cases come from China, and HCC ranks as the second most common cause of death in China (Xie et al., 2020). HCC is usually developed through a gradual process: chronic hepatitis, liver fibrosis, liver cirrhosis, and finally HCC (Kanda et al., 2019). Over the past decade, although many breakthroughs in the treatment of HCC have been made, there is still facing difficulties. The high recurrence rate after surgery and interventional therapy, coupled with the difficulty of chemotherapy and targeted medicines to achieve the desired efficacy, makes the overall survival rate of HCC patients not optimistic, and only about 10% of patients can survive the 5-year survival period (Wang and Lu, 2024). Therefore, there is an urgent need to find new strategies for HCC treatments. The metabolic changes in patients with HCC have garnered significant interest in recent years. Abnormal lipid metabolism has shown strong associations with the incidence and development of HCC in previous studies (Sangineto et al., 2020). For example, scientists have found that HCC is typically characterized by upregulation of genes associated with fatty acids (FAs) synthesis (Budhu et al., 2013). In another study, enhanced lipid synthesis as well as the upregulation of some lipid metabolism-related genes was observed in β-catenin-activated HCCs (Berndt et al., 2019a). Consequently, abnormal lipid metabolism has become a new target for treating HCC.

Normal lipid metabolism involves the uptake, synthesis, and decomposition of FAs, phospholipids, and cholesterol. Synthesized and stored lipids function as energy sources for cells, secondary messengers that transmit information, and involved in biofilm synthesis, thereby maintaining normal cell survival. Abnormal lipid metabolism, which manifests as abnormal FA synthesis and lipid accumulation, is observed in patients with HCC (Bort et al., 2020; Xu et al., 2023). It provides cancer cells with a large amount of energy for proliferation, survival, invasion, and metastasis while facilitating FA-induced reprogramming of lipid metabolism (Hu et al., 2020; Peng et al., 2022; Seo et al., 2020). Moreover, it aids in biofilm synthesis and signaling molecules for cancer treatment response and the tumor microenvironment (Bian et al., 2021). Lipids play an important role in signal transduction and molecular recognition, acting as first (extracellular) and second (intracellular) messengers. Specifically, membrane glycerides and sphingolipids are able to hydrolyze to transduce signals to produce biologically active molecules such as ceramides and sphingosine-1-phosphate (S1P). Steroids (including oxysterols, bile acids (BAs), steroid hormones) and FAs are involved in these processes through direct interactions with receptors (Paul et al., 2022). Among them, C24 ceramide is associated with the aggregation of cancer. The proportion of C24 ceramide in tumor-derived exosomes indicates the tumor dry-like phenotype of glioblastoma. Besides, the loss of ceramide and S1P signaling leads to a decrease in the amount of exosome release (Jia et al., 2024). Thus, regulating lipid metabolism is believed to affect the development of HCC. Several studies have observed that drugs targeting key or rate-limiting metabolic enzymes, metabolites, and drivers of metabolic changes are effective in treating HCC. For instance, the likelihood of HCC in individuals with severe liver disease may be diminished through the use of 3-hydroxy-3-methyl-glutaryl-coenzyme A (HMG-CoA) reductase inhibitors, commonly referred to as statins (Hsiang et al., 2015). The inclusion of statins, as has been noted in numerous clinical trials, extends survival for patients suffering from advanced HCC (Kawata et al., 2001; Shao et al., 2015). Fenofibrate, commonly used to treat hypertriglyceridemia and dyslipidemia, is a fibrillic acid derivative. It plays a role in cancer treatment by regulating related lipid metabolism pathways, such as the PPARα/RXR pathway (La Fountaine et al., 2020; Lian et al., 2018). However, efficacy and safety of these drugs in HCC treatment still require further clinical validation.

**FIGURE 1:**
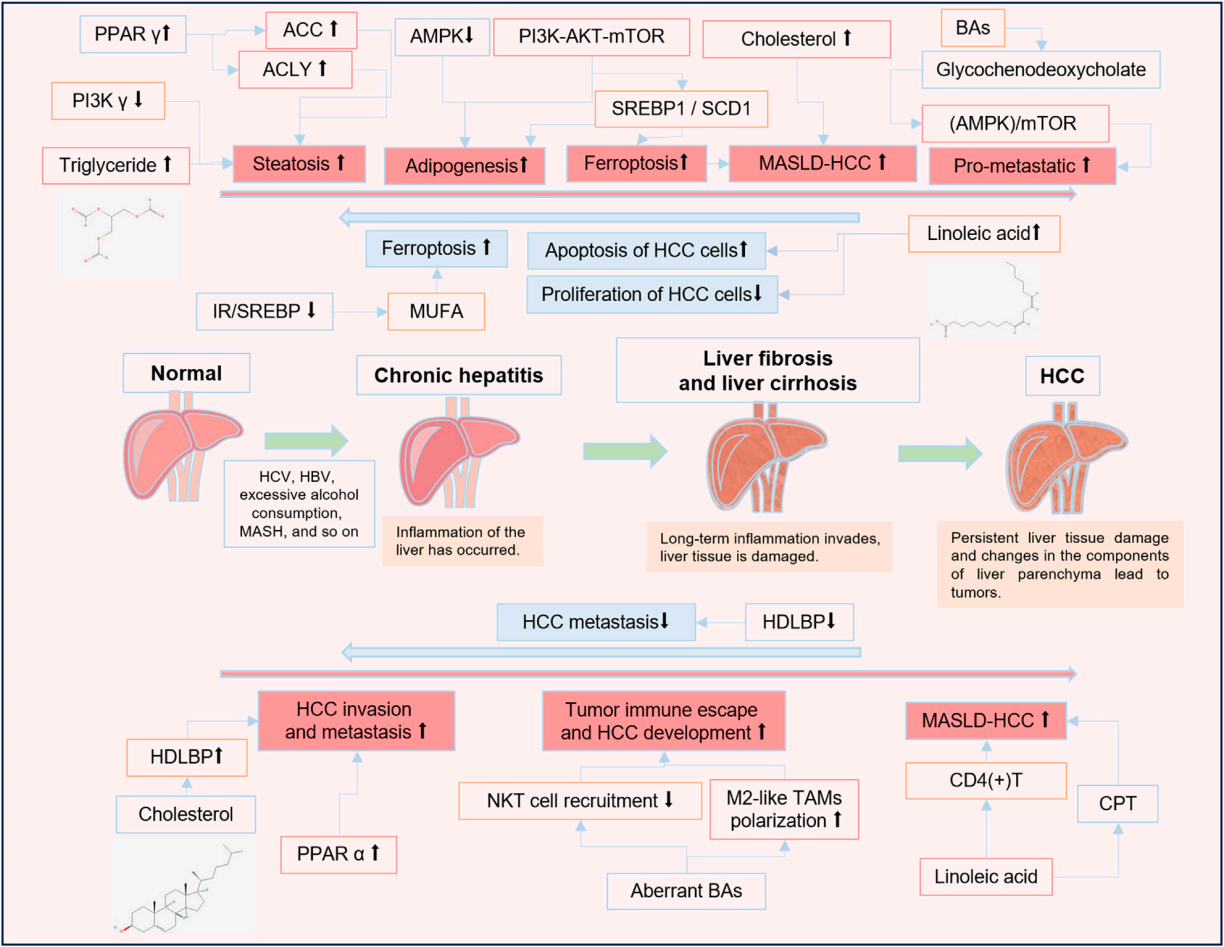
The roles of lipid metabolites and lipid metabolism-related signaling pathways in HCC progression. ↑ An increase in the level or a positive effect. ↓ A decrease in the level or an inhibitory effect. 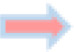 Promoting HCC progression. 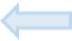 Inhibiting HCC progression. The change in the color of the liver in this figure indicates the progression of the disease, the reddening of the liver indicates inflammation in the liver, and the darkening of the liver indicates fibrosis and tissue damage.

**FIGURE 2:**
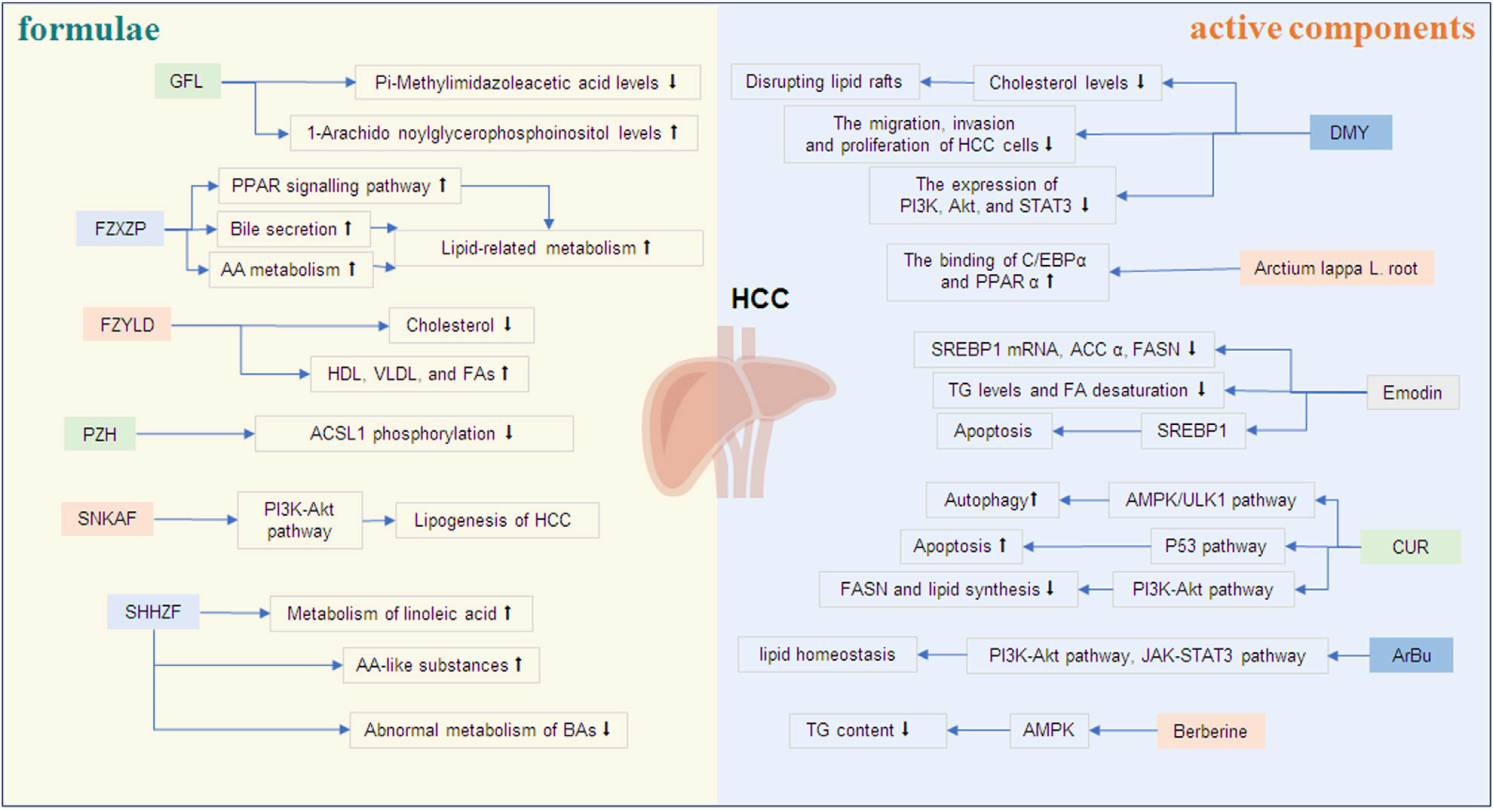
The effects of TCM formulae and active components on lipid metabolism of HCC. ↑ An increase in the level or a positive effect. ↓ A decrease in the level or an inhibitory effect. Note: GFL, Ganfule capsules; FZYLD, Fuzheng Yiliu Decoction; FZXZP, Fuzheng Xiaozheng prescription; PZH, Pien Tze Huang; SNKAF, Sinikangai fang; SHHZF, Shuihonghuazi Formula; DMY, Dihydromyricetin; CUR, Curcumin; ArBu, Arenobufagin.

Formulae and active components of traditional Chinese medicine (TCM) have been used to treat various cancers and metabolic related diseases with good efficacy and safety (Wei et al., 2022). It could regulate metabolic processes by influencing glycolysis, mitochondrial oxidative phosphorylation, glutaminolysis, FA biosynthesis and so on (Wang et al., 2021). Recently, emerging researches revealed that some TCM formulae and active components exert therapeutic effects in patients with HCC by regulating lipid metabolism and the products of lipid metabolism or affecting the pathways of lipid metabolism. However, the unclear action characteristics and therapeutic mechanisms limit the precise application of TCM formulations in clinic. Therefore, we searched the English articles from PubMed in the last 5 years for the keywords: traditional Chinese medicine, hepatocellular carcinoma, and lipid metabolism, hepatocellular carcinoma and linoleic acid, hepatocellular carcinoma and cholesterol, hepatocellular carcinoma and arachidonic acid, hepatocellular carcinoma and triglycerides, hepatocellular carcinoma and lipoprotein, traditional Chinese medicine and linoleic acid, traditional Chinese medicine and cholesterol, traditional Chinese medicine and arachidonic acid, traditional Chinese medicine and triglycerides, traditional Chinese medicine and lipoprotein. Then, we collected and summarized them, so as to describe the changes and the roles of lipid metabolites in HCC progression (Figure 1), and discuss the effect of TCM formulae and active components in the treatment of HCC based on their regulation on abnormal lipid metabolism (Figure 2).

## 2 Abnormal lipid metabolism in HCC

### 2.1 Abnormal FAs levels in HCC

Normally, FAs can be categorized into two primary groups based on their molecular structure: unsaturated fatty acids (UFAs) and saturated fatty acids (SFAs). Within the UFAs category, there are further distinctions made between polyunsaturated fatty acids (PUFAs) and monounsaturated fatty acids (MUFAs). Among PUFAs, specific types include linoleic acid, linolenic acid, and arachidonic acid (AA), et al. It has been observed that abnormal levels of linoleic acid are prevalent in individuals diagnosed with HCC (Cai et al., 2020). Researchers have discovered that the concentration of linoleic acid in the portal vein is reduced in patients with HCC compared to healthy individuals (Liu J. et al., 2022). Vlock EM and colleagues used a mouse model of lean MASH-HCC to determine the changes of linoleic acid levels in HCC. The findings indicated these mice model had a decrease in linoleic acid levels; however, in mice fed choline-deficient, high trans-fat, sucrose, and cholesterol diets, levels increased over time (Vlock et al., 2020). In contrast, AA, another metabolite belonging to PUFA, was elevated in HCC. One experiment used the metabolomics to distinguish HCC patients from those without cancer, researchers discovered that the concentration of AA was notably elevated in HCC cohort relative to the control group (Jee et al., 2018). Additionally, some other FAs were also discovered to be abnormal in HCC. Researchers examined the association between free FAs and advanced liver fibrosis or HCC in hispanics in South Texas who have high rates of HCC. They found that HCC has showed a significant association with low expression of some types of FAs, such as very long chain (VLC) SFAs, odd chain SFAs, and VLC n-3 PUFAs (Jiao et al., 2021).

### 2.2 Abnormal cholesterol levels in HCC

Cholesterol is an essential lipid for maintaining cellular homeostasis synthesized mainly in the liver (Luo et al., 2020). A study aimed to compare the metabolic signature between MASH and MASH-HCC patients. The results showed that compared with the MASH group, BAs metabolism and cholesterol metabolism were significantly upregulated in MASH-HCC group (Ahmed et al., 2022). Plasma cholesterol in 132 HCC patients and 287 patients without HCC cirrhosis was studied by Venturini et al. and the result suggested that the enhanced cholesterol biosynthesis led to a rise in plasma cholesterol of patients with cancer (Venturini et al., 1999). Besides, researchers assessed the extent of FA synthesis in the liver, as well as levels of triglycerides and cholesterol to investigate the role of unconstrained lipogenesis in human HCC. The findings revealed that the synthesis of FAs, as well as triglyceride and cholesterol levels, was significantly elevated in HCC compared to the surrounding liver tissue (Calvisi et al., 2011). Additionally, it was discovered that HCC patients with shorter survival times exhibited higher serum cholesterol levels (Li et al., 2023a). However, it has also been reported to the contrary that high cholesterol is associated with a low risk of HCC. In one experiment, underwent health screenings in 2009, a total of 8,528,790 patients were analyzed using Cox regression to investigate the hazard ratios for HCC. The findings indicated that as levels of low-density lipoprotein cholesterol and total cholesterol increased, the incidence of HCC progressively decreased. Compared to individuals in the lowest quartile of total cholesterol, those with higher levels of total cholesterol were associated with a reduced risk of developing HCC (Cho et al., 2021).This result suggests that the relationship between HCC progression and cholesterol levels still needs to be further studied.

Bile acids (BAs) play a crucial role in cholesterol metabolism, with the conversion of cholesterol into BAs being the most important metabolic pathway. At the same time, about 40% of the cholesterol in the body of a normal person is converted into BA every day. An enhanced BA pool has been observed in patients with MASH-associated HCC (Conde de la Rosa et al., 2021). The quantification and analysis of 35 types of BAs in the pre- diagnostic sera of 100 patients with HCC and 100 healthy individuals in China revealed the levels of conjugated primary BAs are markedly elevated (Thomas et al., 2021). An increased risk of HCC is associated with higher BAs concentrations, particularly in conjugated primary BAs (Petrick et al., 2020). One study observed an association between the elevated levels of major circulating BAs and the increased risk of HCC through analyzing 233 cases of HCC. With increasing concentrations of BAs, the BAs profile shifted towards a higher proportion of taurine-conjugated BAs, suggesting the progression of HCC and early metabolic changes in BAs metabolism (Stepien et al., 2022). BA synthesis and transport are regulated by farnesoid X receptor (FXR). It has been reported that in mice lacking the FXR, both hepatic and serum levels of BAs are elevated. The accumulation of BAs in these FXR-deficient mice led to a spontaneous development of HCC in nearly 90% of cases (Kim et al., 2007). However, not all studies found that BAs was elevated. In one study, scientists investigated BAs levels and genes associated with BAs homeostasis in 37 patients with HCC in both tumor-adjacent and cancer tissues, finding a 36% reduction in total BAs (Chen W. et al., 2023). In another study involving 348 patients with chronic liver disease and 396 patients with HCC, the findings revealed that the total BAs levels in HCC patients, both prior to and following propensity score matching analysis, were lower compared to those with persistent liver diseases. Additionally, there was a significant reduction in the total BAs levels observed in HCC patients (Dai et al., 2024).

### 2.3 Abnormal triglycerides levels in HCC

An increase in the levels of saturated triglycerides (TG) had been reported in HCC. Using untargeted metabolomics and lipidomics to study the biomarkers of HCC in patients with cirrhosis, researchers established that TG levels were significantly raised in blood of HCC cases (Rashid et al., 2023). To better comprehend the molecular pathogenesis of lipid metabolism levels, separate tests of blood from patients with HCC, healthy people, and patients with chronic liver disease was used. The results indicated that several predominantly saturated TGs in the blood showed a sustained increase in HCC trajectories (Ismail et al., 2020). Excessive carbohydrates being converted to TG or an increased delivery of TG to the liver causes excessive accumulation of fat in the liver (Lee J. et al., 2017). Experimental results have shown that lipid deposition within the tumor is a characteristic feature of HCCs, with the accumulation of diglycerides and TG being observed in the tumor tissue of mice (Haberl et al., 2021). A study established mouse models of lean and obese MASH-HCC to compare their progression toward MASH-HCC. The results revealed that TG levels in both groups had elevated (Hymel et al., 2022). In another study, the progression of high-fat diet-induced MASLD-MASH-HCC and elevated cholesterol and TG levels were also detected in the mice (Tessitore et al., 2016). Scientists used protein intensity profiles of 11 human HCCs to parameterize tumor-specific kinetic models of cellular lipid metabolism. They found that compared to the HCC tumor-adjacent tissue, TG content in HCC was significantly higher (Berndt et al., 2019b). Logistic regression analysis showed that the TG levels were an separate risk factor for the incidence of HCC (Ali et al., 2022).

### 2.4 Abnormal lipoprotein and apolipoprotein levels in HCC

Abnormal lipoprotein metabolism is also closely associated with tumorigenesis. There are some typical human lipoproteins: low-density lipoproteins (LDL), very-low-density lipoproteins (VLDL), high-density lipoproteins (HDL) and chylomicrons (CM). Increased secretion of lipoprotein lipase from the tumor cells into the peripheral blood has been observed in patients with HCC. A significant decrease in the serum VLDL levels, and a significant increase in the serum LDL levels have been observed in patients with HCC (Zuo et al., 2021). Apolipoprotein A1 (ApoA-1) is an indispensable protein in the synthesis of HDL. Scientists have found that ApoA-1 levels correlated with the degree of malignancy in HCC. Patients with HCC and tumor recurrence exhibited significantly reduced serum ApoA-1 levels. Additionally, higher serum ApoA-1 levels were observed in patients with reduced levels of circulating tumor cells (Ma X. L. et al., 2016). Analysis of prospective data collected by the Türkiye HCC multi-agency cooperative working group revealed that low HDL and high LDL levels showed significant correlations with an increase in maximum tumor diameter. Elevated LDL levels and reduced HDL levels in plasma are significantly associated with a more aggressive HCC phenotype, especially when these conditions occur together. This combination is also linked to an increased risk of death (Akkiz et al., 2021). Lipoprotein (a) [Lp (a)] is a molecule bound to apolipoprotein (a) (Tasdighi et al., 2024). Scientists assessed the relationship between HCC and Lp(a) levels in serum by Kaplan-Meier curves and log-rank tests. Comparing data from healthy individuals and HCC patients, Lp(a) levels in HCC patients were found to be significantly lower than in healthy individuals. In addition, there is an association between HCC recurrence and Lp(a) levels, HCC patients with low Lp(a) levels have a higher recurrence rate and shorter survival than those with high Lp(a) levels (Gao et al., 2018).

## 3 Abnormal lipid metabolism affects HCC progression

### 3.1 Affecting cell proliferation and apoptosis

The recognition of abnormal cholesterol metabolism in HCC has led to an increase in the focus on the effects of it in HCC. In an experiment in which researchers fed mice with high fat/cholesterol, it developed HCC and decreased tryptophan metabolism associated with the gut microbiota, correlation analysis showed that dysbiosis of gut microbiota in MASLD-HCC was associated with cholesterol levels. Germ-free mice gavaged with stools from mice fed high fat/cholesterol manifested hepatic lipid accumulation, inflammation and cell proliferation. Scientists have shown that dietary cholesterol-regulated microbiota promotes MASLD and hepatocyte proliferation by inducing metabolite alterations, thereby promoting cholesterol-induced MASLD-HCC formation (Zhang et al., 2021). Not only cholesterol, but also some FAs can influence the cell proliferation and apoptosis in HCC. Scientists have studied the functional importance of linoleic acid in HCC cell lines. They found that linoleic acid could significantly inhibit the proliferation and induce the apoptosis of HCC cells (Liu J. et al., 2022). Ferroptosis, characterized by lipid peroxidation and iron accumulation that is strongly associated with HCC progression, is an emerging iron-dependent programmed cell death modality (Yang et al., 2024). Accumulating evidence suggests that activation of ferroptosis may prevent HCC cell proliferation (Nie et al., 2018). Lipidomic analysis has shown that promoting the selective uptake of MUFAs could induce an increase in phosphatidylcholine and phosphatidylethanolamine levels in HCC cells, which induces resistance to lipid peroxidation and ferroptosis (Li et al., 2023b). Meanwhile, in another experiment, scientists found that downregulation of IR/SREBP axis-mediated MUFA synthesis could promote ferroptosis in HCC, thus inhibiting HCC (Yuan et al., 2024).

### 3.2 Affecting tumor invasion and metastasis

The lipid transporter high-density lipoprotein-binding protein (HDLBP) is clinically relevant to tumor metastasis in patients with HCC. Cholesterol-induced HCC metastasis needs HDLBP. Scientists suggested that cholesterol-induced HDLBP promoted HCC metastasis and invasion through BRAF-dependent epithelial-mesenchymal transition (EMT) signaling. Further studies have shown that knockdown or overexpression of HDLBP significantly inhibited or enhanced the metastasis, invasion and EMT of HCC cells, respectively (Yuan et al., 2022). Known as an indispensable adaptive survival mechanism, autophagy was speculated by researchers that may play a role in HCC metastasis by promoting the metastatic colonization of HCC cells (Peng et al., 2013). Scientists found that glycochenodeoxycholate, which is important for the synthesis of BAs, mediated autophagy in HCC cells through the AMP-activated protein kinase (AMPK)/mTOR signaling pathway, exhibiting a pro-metastatic effect both *in vitro* and *in vivo* (Gao et al., 2019).

### 3.3 Affecting the tumor microenvironment

The tumor microenvironment (TME) is an intricate network, predominantly consisting of cancer cells, vascular endothelial cells, penetrating immune cells, carcinoma-linked fibroblasts, and adipose tissues. This milieu is vital for the advancement and multiplication of HCC (Giraldo et al., 2019; Li et al., 2007). Immune cell infiltration including CD4(+) T cells, CD56 NK cells, macrophage and so on (Liu Y. et al., 2022; Bao et al., 2019). In the TME of HCC, the lipid profile of macrophages changes (Yeung et al., 2015). Previous studies have shown that activating M2 macrophage polarization could promote HCC cell invasion and migration (Huang et al., 2023). Aberrant BAs metabolism has been found to modulate the TME by preventing natural killer T (NKT) cell recruitment and increasing M2-like tumor-associated macrophages (TAMs) polarization, thereby promoting tumor immune escape and HCC development (Xia et al., 2022). Immune responses requires CD4(+) T cells to against pathogens and cancer cells (Malyshkina et al., 2023). Previous studies have suggested that the development of HCC may be related to the contribution of CD4(+) T cells loss to the impact. Scientists suggested that linoleic acid can disrupt mitochondrial function and cause more oxidative damage, producing more mitochondria-derived reactive oxygen species (ROS), which could promote the selective loss of intrahepatic CD4(+) T cells, accelerated MASLD-facilitated HCC (Ma C. et al., 2016). Moreover, the long chain of FA is transported from the cytoplasm into the mitochondria by the carnitine palmitoyl transferase (CPT) system and β oxidation occurs. In HCC, induction of the CPT gene increases ROS and leads to apoptosis of CD4(+) T cells. Linoleic acid, on the other hand, can induce CPT gene expression. This suggests that upregulation of the CPT gene by linoleic acid induces CD4(+) T cell apoptosis, thereby promoting HCC development (Brown et al., 2018). This seems to be contrary to the results of the previous experiments, perhaps due to the different models of research, and the role of linoleic acid in serum and cells may be different.

## 4 Signaling pathways affecting lipid metabolism in HCC

### 4.1 PPAR related signaling pathways

Peroxisome proliferator-activated receptor (PPAR) is a FA-activated transcription factor. It has three PPAR subtypes: PPAR α, PPAR γ, and PPAR β/δ. PPAR can trigger FA biosynthesis, is the “bridge” between FA imbalance and the maintaining of cancer cell stemness (Feng et al., 2022). PPAR γ is a key transcription factor related to lipid metabolism. The increased expression of PPAR γ, ATP citrate lyase (ACLY) and acetyl-CoA carboxylase (ACC) are associated with steatosis of HCC (Ning et al., 2022). Besides, PPAR γ regulates lipid synthesis by upregulating the transcription of lipid synthesis enzymes (ACLY, ACC, and FASN) (Desvergne et al., 2006). Numerous studies have established the role of PPAR α in lipid and lipoprotein metabolism of HCC, too. After the activation of PPAR α, the expression of its downstream target genes in liver samples and the level of enzymes involved in the β oxidation of FAs increased. And high PPAR α expression in HCC is associated with poor prognosis (Chen et al., 2021). One study showed that activation of PPAR α in rat HCC cell lines led to reduced triglyceride concentrations in liver, plasma, and very low-density lipoprotein (König and Eder, 2006). Besides, increased expression of PPAR α enhances intracellular oxidative stress, thereby promoting HCC invasion and metastasis (Lin et al., 2021). Retinoid X receptor α (RXR α) is a nuclear receptor for retinoid (Sakai et al., 2022). It has been found a dysregulation of both RXR α function and FA metabolism in HCC (Castro-Gil et al., 2022). Scientists believe that the PPAR α/RXR α signaling pathway can be involved in metabolic diseases in humans by regulating oxidative stress, lipid metabolism, and inflammatory pathways (Yang et al., 2022).

### 4.2 PI3K/AKT related signaling pathways

Phosphatidylininosine-3 kinases (PI3Ks) are promising medicine targets for therapy of HCC, with four PI3Ks: PI3K α, PI3K β, PI3K δ, and PI3K γ. Studies have shown that loss of PI3K γ reduces tumor development of obesity-promoted HCC through multiple cell types and mechanisms, including steatosis (Becattini et al., 2021). Researchers found that the PI3K-AKT-mTOR pathway can activate lipid synthesis and promote NAFLD-HCC progression (Chen et al., 2019). The oncogenic activation of PI3K-AKT-mTOR signaling can activate sterol regulatory element-binding protein 1 (SREBP1), a central transcription factor that regulates lipid metabolism, which then affects adipogenesis in HCC, thereby killing cancer cells. At the same time, stearoyl-coenzyme A desaturase-1 (SCD 1) is the transcriptional target of SREBP1. Hyperactive mutations in PI3K-AKT-mTOR signaling protect cancer cells from oxidative stress and ferroptosis through SREBP1/SCD 1-mediated lipogenesis. Besides, the AKT/mTOR pathway can regulate SREBP1c and thus regulate *de novo* adipogenesis (Yi et al., 2020; Yecies et al., 2011).

### 4.3 AMPK related signaling pathways

AMP-activated protein kinases (AMPKs) are key cellular energy sensors (Li et al., 2011). The AMPK signaling pathway is related to lipid synthesis in HCC, and inhibition of the AMPK pathway can induce lipid synthesis in HCC. This is mainly done by regulating its downstream genes, ACC1 and ACLY (Zhang Y. et al., 2022). Previous studies demonstrate that AMPK could inhibit the transactivity of SREBP1. One study found that the interaction between the HCC-associated protein TD26 and the truncated nuclear SREBP1 form (nSREBP1) could block AMPK-mediated SREBP1 inhibition, resulting in increased lipogenesis and enhanced HCC progression (Wang et al., 2018).

### 4.4 Others

Exosomes are important tools for intracellular communication and have a significant impact on HCC progression and metastasis. A study investigated the changes of lipid classes in HCC derived exosomes. The researcher collected the exosomes from plasma in patients with cirrhosis (31 with HCC and 41 without HCC). The results demonstrated that some lipid species, such as phosphatidylcholines (18:3e/22:4), phosphatidylcholines (16:1e/22:6), TG (18:0/14:0/16:0), TG (25:0/16:0/17:0), monogalactosyldiacylglycerols (16:0/21:6), et al., were detected in the majority of HCC exosomes but in none of the non-HCC exosomes. Further pathway analysis showed that glycerophospholipid metabolism, retrograde endocannabinoid signaling and ferroptosis were associated with the changes in lipid composition in HCC exosomes (Sanchez et al., 2021).

## 5 TCM formulae and active components alleviates HCC progression by regulating lipid metabolism

Dysregulated lipid metabolism has been identified as a critical driver of HCC progression. Notably, emerging research reveals that specific TCM formulae and active components may exert anti-HCC activity by regulating lipid metabolism through targeting aberrant cholesterol synthesis, fatty acid oxidation, and lipid droplet accumulation (Tables 1, 2; Figure 2). This metabolic regulation not only suppresses HCC growth but also enhances the efficacy of biological agents, offering a dual therapeutic advantage.

**TABLE 1:** The composition and effect of TCM formulae.

Formulae of Chinese medicine	Composition	Preparation	Model	Administration	Effect	References
Ganfule capsules	*Codonopsis pilosula* (Franch.) Nannf, *Trionyx sinensis* Weigmann, *Paris polyphylla* var.chinensis (Franch.) H.Hara*, Atractylodes macrocephala* Koidz*, Astragalus mongholicus* Bunge*, Citrus reticulata* Blanco*, Steleophaga plancyi*(Boleny)*, Rheum palmatum* L*, Prunus persica* (L.)Batsch*, Scutellaria barbata* D.Don*, Patrinia villosa* (Thunb.)Dufr*, Poria cocos* (Schw.)Wolf*, Coix lacryma-jobi* L.var.ma-yuen *(Roman.)Stapf, Curcuma longaL, Biancaea sappan* (L.)Tod*, Ostrea gigas* Thunberg*, Artemisia scoparia* Waldst.et Kit, *Akebia quinata*(Thunb.)Decne, *Cyperus rotundus* L, *Aquilaria sinensis*(Lour.)Gilg, *Bupleurum chinense* DC. (Ke et al., 2022)	The active components of GFL capsules(specification: 0.5 g/capsule, production batch number: 20180505, Kamp Pharmaceutical Co., Ltd) were analyzed by UPLC-Q-TOF/MS. 50 mg of the capsule contents powder was weighed, dispersed in 10 mL of methanol-water mixed solvent (1:1, v/v) by ultrasonic-assisted extraction technology (working frequency 40 kHz, duration 30 min), centrifugation (relative centrifugal force 401×g, for 15 min) to obtain a clear extract, and 3 μL of supernatant was injected for analysis. The separation capsule step was performed on a Waters ACQUITY UPLC BEH C18 reversed-phase column (100 mm × 2.1 mm, particle size, 1.7 μm) with a constant temperature of 35°C in positive ion mode (POS) and negative ion mode (NEG)	Nude murine model induced by HepG2 cell	The mice were gavaged with 0.2 mL of GFL (7.4 g/kg) every day, continuous administration for 2 weeks	• ↑1-Arachido noylglycerophosphoinositol • ↓Pi-Methylimidazoleacetic acid	Xu et al. (2022)
Fuzheng Yiliu Decoction	*Astragalus mongholicus* Bunge*, Ligustrum lucidum* W.T.Aiton*, Dioscorea polystachya* Turcz*., Rhinacanthus nasutus* (L.) Kurz.	*Astragali Radix* and *Ganoderma* made in Fuzhou Huichun Chinese medicine Yinpian Factory Co. Ltd. (batch number: 10010818 and 10010605), *Fructus ligustri lucid* (Shanghai Leiyun Shang decoction piece, batch number 20110101) and *Dioscoreae Rhizoma* (Anhui Wansheng Traditional Chinese Medicine, batch number 110108). After ultrafine grinding (through 60 mesh sieve), the medicinal materials were accurately weighed and matched according to the mass ratio of 2:2:1:1 to form a compound system with a total mass of 450 g (including 150 g of *Astragali Radix*, 150 g of *Ganoderma*, 75 g of *Fructus ligustri lucid* and 75 g of *Dioscoreae Rhizoma*). Preparation process: In the first decoction stage, the mixed herbs were soaked with 6 times (2.7 L) of deionized water at room temperature for 30 min, and then the decoction was carried out by gradient heating method (after boiling, the temperature was maintained for 20 min), and 1.5 L of the initial decoction liquid was collected. The second decoction was re-extracted with the same process parameters, and the two decoctions were refined through a double-layer microporous filter membrane (0.45 μm), and then concentrated to a final volume of 1 mL under reduced pressure at 60°C, and the concentration was confirmed to be equivalent to 0.45 g/mL of raw medicinal materials by quality control testing	Orthotopic transplantation rat model induced by HepG2 cells	The rats were gavaged with 0.1 mL of FZYLD every day, continuous administration for 2 weeks	• ↓UFAs and cholesterol • ↑HDL, VLDL, LDL and FAs	Zhang et al. (2022b)
Fuzheng Xiaozheng prescription	*Astragalus mongholicus Bunge, Trionyx sinensis Weigmann, Sparganium stoloniferum* (Buch. - Ham. ex Graebn.) Buch. - Ham. ex Juz*., Curcuma aromatica* Salisb*., Prunus persica* (L.) Batsch*, Carthamus tinctorius* L*., Angelica sinensis* (Oliv.) Diels*, Glycyrrhiza uralensis* Fisch. ex DC.	The main herbs in FZXZP are provided by Beijing Tong Ren Tang Science and Technology Development Co., Ltd. (Beijing). Differentiated treatment strategies were implemented: plant medicinal materials were infiltrated with deionized water at room temperature (30 min), and animal-derived medicinal materials were treated with extended pretreatment cycle (40–50 min). Referring to the standard decoction process (water added: 10 times the volume of the material), the initial decoction stage: after the animal medicinal materials were decocted for 20 min, plant medicinal materials were added for co-decoction, and the extraction was maintained at a simmer for 30 min After the liquid formulae is collected by the residue filtration system, the circular extraction process (1–2 repeats) is adopted. Multiple batches of extracts were combined, refined by a three-stage gradient filtration system, concentrated to a density of 1.26 g/cm^3^ under vacuum decompression at 60°C, and stored in the cold chain at 4°C. The whole process follows the specifications of the Chinese Pharmacopoeia	HCC rat model induced by diethylnitrosamine	The rats were gavaged with 0.1 mL of FZXZP (12.6 g/kg, 6.3 g/kg and 25.2 g/kg) every day, continuous administration for 14–18 weeks	• ↑Lipid-related metabolism • Regulating some lipid-related metabolism such as AA, linoleic acid and retinol • ↑The biosynthesis of steroid hormones	Liu et al. (2022c), Li et al. (2022)
Pien Tze Huang	*Pana.c notoginseng*(Burk.) F.H.Chen*, Abelmoschus moschatus* Medik.*, Bos taurus domesticus* Gmelin*,* Snake gall^a^	The pharmaceutical product PZH (batch 1607039) was manufactured and certified by Zhangzhou Pien Tze Huang Pharmaceutical Co., Ltd., (located in Zhangzhou, China, under CFDA approval number Z35020243)	HCC mice model introduced by Hepa1-6 cells	The mice were gavaged with PZH (234 mg/kg) every day, continuous administration for 3 weeks	• ↓Phosphorylation of ACSL1 associated with FA biosynthesis/degradation pathway	Lin et al. (2023)
Sinikangai fang	*Hedyotis diffusa* var. longipes Nakai*, Eupolyphaga sinensis* Walker*, Scutellaria barbata* D.Don*, Solanum nigrum* Acerbi ex Dunal*, Akebia trifoliata* (Thunb.) Koidz*., Bupleurum chinense* DC.*, Paeonia lactiflora* Pall*., Glycyrrhiza glabra* L. *Codonopsis pilosula* (Franch.) Nannf.*, Atractylodes macrocephala* Koidz.*, Coix lacryma-jobi* var. ma-yuen (Rom.Caill.) Stapf*, Poria cocos* (Schw.) Wolf*, Prunus persica* (L.) Batsch*, Cremastra appendiculata* (D. Don) Makino.	The samples were completely immersed in quantitative distilled water and then allowed to stand for 30 min, and then gradually heated to boiling (100°C ± 1°C) to complete two independent decoction procedures. Immediately after each decoction, the filtrate is collected by means of a double-layer sterile gauze for solid-liquid separation. After combining the two clarification solutions, they were concentrated and stored at a constant temperature of 4°C ± 0.5°C	HCC xenograft mouse model introduced by MHCC-97H cells	The mice were gavaged with SNKAF (15.3 g/kg, 30.6 g/kg and 61.2 g/kg) every day, continuous administration for 15 days	• Regulating the PI3K/Akt pathway	Guo et al. (2022a)
Shuihonghuazi Formula	*Persicaria orientalis* (L.) Spach*, Imperata cylindrica* (L.) Raeusch.*,* Ophicalcitum^b^ *, Coix lacryma-jobi* var. ma-yuen (Rom.Caill.) Stapf	This formula was prepared in the laboratory in strict accordance with the standards of the 2020 edition of the Chinese Pharmacopoeia, and Qinhuangdao Taijihuan Nano Technology Co., Ltd. was entrusted to implement the transformation of nanoscale particle preparations	HCC rats model induced by diethylnitrosamine	The rats were gavaged with SNKAF (757 mg/kg, soluble in PEG-400) every day, continuous administration for 9 weeks	• ↑The absorption and utilization of linoleic acid and oleic acid • ↑AA-like substances • ↓The abnormal metabolism of BAs	Bao et al. (2017)

Notes: The standard names of some medicine are not available and are explained here.

^a^ The dry gallbladder of a snake.

^b^ This product is a metamorphic rock type rock, serpentine marble. Main contains (CaCO3).

↑ An increase in the level or a positive effect.

↓ A decreased in the level or an inhibitory effect.

**TABLE 2:** The effect of the active components from TCM.

TCM active components	Chemical structure	Model	Administration	Effect	Target	References
Dihydromyricetin	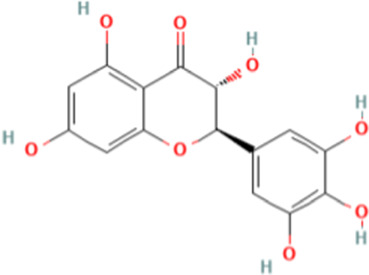	HepG2, Hep-3B, 97H, SMMC-7721, Sk-Hep1, and Huh7 cells	DMY (0, 20, 40, 80, 160, 320 μM)	• ↓EGFR and its downstream pathways. • ↓Cholesterol level • ↓The expression of lipid raft markers	CAV1, FLOT1, EGFR, PI3K, Akt and STAT3	Zhang et al. (2023)
Arctigenin	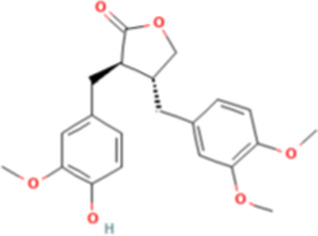	HepG2 and Hep-3B cells	Arctigenin (20 μM)	• ↑The binding between C/EBPα and PPARα • ↓gankyrin	C/EBPα, PPARα	Sun et al. (2018)
Emodin	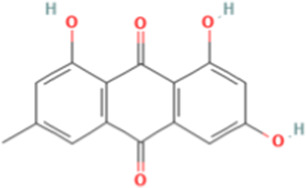	Bel-7402 cell	Emodin (100 μM)	• ↓The TG levels and FA desaturation • ↓SREBP1 and acid metabolism-related proteins (ACC α, FASN, and stearoyl-CoA A desaturase D)	SREBP1	Yang et al. (2019)
Curcumin	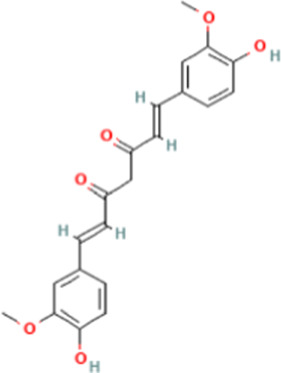	HepG2 cells	CUR (0, 20, 40, and 60 μM)	• ↑HepG2 cell apoptosis • ↑HepG2 autophagy	P53 and AMPK/ULK1 pathway	Chen et al. (2022)
		H22 bearing mice	The mice were gavaged with CUR (25 mg/kg), continuous administration for for 3 weeks	• ↓FASN and lipid synthesis	PI3K/Akt pathway	Man et al. (2020)
Arenobufagin	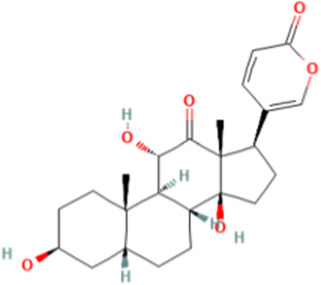	HepG2 cells	ArBu (1.1 ng/mL and 3.3 ng/mL)	• ↓The expression of 65 differential proteins related to lipid metabolism, cell apoptosis, and autophagy	PI3K/Akt and JAK-STAT3 pathway	Zhao et al. (2020)
Berberine	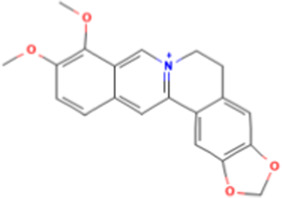	HepG2 cells	Berberine (15 μM)	• ↓TG content	AMPK	Cao et al. (2018)

Notes: ↑ An increase in the level or a positive effect. ↓ A decreased in the level or an inhibitory effect.

### 5.1 TCM formulae

Ganfule capsules (GFL), approved by the National Medical Products Administration of China, is used as a complementary alternative therapy for the treatment of HCC. It comprised of 21 herbs, including *Codonopsis pilosula* (Franch.) Nannf, *Trionyx sinensis* Weigmann*,* et al. (Ke et al., 2022). Xu F et al. demonstrated that GFL exerted anti-HCC effects by regulating lipid metabolism. Metabolomics identified 426 metabolites and 343 metabolite variants in the positive and negative ion patterns after GFL treatment in a nude murine model of HepG2 cell injection. These genetic variations are potentially associated with lipid metabolic pathways, and the metabolites derived from these pathways have been implicated in mediating the protective actions of GFL against HCC. For example, GFL can increase the 1-Arachido noylglycerophosphoinositol levels while decreasing Pi-Methylimidazoleacetic acid levels (Xu et al., 2022).

Fuzheng Yiliu Decoction (FZYLD) consists of four herbs: *Astragalus mongholicus* Bunge*, Ligustrum lucidum* W.T.Aiton*, Dioscorea polystachya* Turcz*.,* and *Rhinacanthus nasutus* (L.) Kurz*.* Scientists have been using FZYLD in clinical practice for the treatment of liver tumors for many years, and it can improve clinical symptoms, slow down physical weakness after chemotherapy or radiotherapy, improve the quality of life, and prolong the survival time of cancer patients (Chen et al., 2014). In recent years, studies have found that FZYLD could also ameliorate energy and lipid metabolism disorders. Zhang Hongcheng established a rat HCC model to observe the therapeutic effect of FZYLD. They found that the levels of endogenous serum metabolites and cholesterol decreased, whereas those of HDL, VLDL, and FAs significantly increased when the rats in the HCC model group received FZYLD. Furthermore, the serum UFA levels in the HCC group exhibited a significant reduction after treatment (Zhang H. et al., 2022).

Fuzheng Xiaozheng prescription (FZXZP), derived from the famous decoction powder recorded in the Book of *Wenyilun* during the Ming Dynasty, effectively improves liver function and has beneficial effects on patients with HCC (Liu et al., 2022c). In a rat HCC model induced by diethylnitrosamine, scientists found that FZXZP administration significantly inhibited the progression of HCC in rats. Then, they performed microarrays of circRNA, miRNA and mRNA to further explore the mechanism of action. After a series of screening, they established the competing endogenous RNA networks. Go and KEGG analysis showed that FZXZP promoted lipid-related metabolism by activating the PPAR signaling pathway, AA metabolism, and bile secretion (Li et al., 2022). In another study, researchers also found FZXZP improved the pathological characteristics of HCC rats. Moreover, FZXZP influenced lipid metabolic processes by managing substances like AA, linoleic acid and retinol, as well as increased the production of steroid hormones (Liu et al., 2022d).

Pien Tze Huang (PZH), a formula of Chinese patent medicine, has been used for more than 500 years in China and Southeast Asia to treat various inflammation-related diseases such as hepatitis (Liu et al., 2020). It has also been widely employed in clinical settings for treating various human malignancies, including HCC (Yan et al., 2023). PZH has been approved by the National Medical Products Administration of China for the clinical trial of medicines in advanced primary liver cancer (Lin et al., 2023). A study used PZH to treat Hepa1-6 mice, and the results demonstrated that PZH significantly inhibited xenograft tumor growth. Moreover, a four-fold inhibition of ACSL1 phosphorylation, which is associated with FA biosynthesis/degradation pathway, has been observed in PZH-treated mice (Lin et al., 2023).

Sinikangai fang (SNKAF) decoction is a formula that has been widely used for the treatment of HCC in China. Guo et al. used 4-week-old BALB/c nude mice to construct an HCC xenograft mouse model. They revealed that the anti-cancer effect of SNKAF on HCC was related to cell proliferation and apoptosis, and its action pathway involved the PI3K/Akt pathway axis. SNKAF enables PI3K to induce the activation of the core signaling kinase Akt, which controls a number of downstream effector molecules and drives protein and lipid synthesis and cell development (Guo W. et al., 2022).

Shuihonghuazi Formula (SHHZF), a formula of Chinese medicine, is often used in the treatment of HCC. It consists of four herbs: *Persicaria orientalis* (L.) Spach*, Imperata cylindrica* (L.) Raeusch.*,* Ophicalcitum^b^ *,* and *Coix lacryma-jobi* var. ma-yuen (Rom.Caill.) Stapf. A study used diethylnitrosamine induced rats as HCC animal model, its results showed that SHHZF promoted the absorption and utilization of linoleic acid and oleic acid, increased the content of AA-like substances, translation of phosphatidylethanolamine to phosphatidylcholine, metabolism of linoleic acid, and inhibited the of abnormal metabolism of BAs in rats with HCC (Bao et al., 2017).

Overall, the above studies showed the positive effect of these formulae in the regulation of abnormal lipid metabolism in HCC. However, the specific targets and signaling pathway by which these formulae regulate lipid metabolism remain insufficiently explored in some studies. Moreover, every formula contains multiple active components. Current research has not fully elucidated which specific components drive lipid metabolism regulation. Additionally, whether the different preparation methods of these formulae will affect the effect of lipid metabolism regulation is still obscure. Therefore, the specific mechanism of these formulae on the regulation of abnormal lipid metabolism in HCC is still worth conducting further research.

### 5.2 TCM active components

Dihydromyricetin (DMY) is the major flavonoid in *Ampelopsis grossedentata* (Hand.-Mazz.) W.T.Wang and its pharmacological action has attracted increasing attention in recent years (Guo et al., 2019). The researchers gave HCC cells different concentrations of DMY (0, 20, 40, 80, 160, 320 μM) and observed their effects. The result showed that DMY inhibited EGFR and its downstream pathways by lowering the cholesterol levels, thereby disrupting lipid rafts, which resulted in the inhibition of HCC. Pharmacological experiments revealed that DMY inhibited the migration and invasion of HCC cells and reduced cholesterol levels. At the same time, it also inhibited the proliferation of HCC cells. Furthermore, DMY downregulated the expression of lipid raft markers (CAV1 and FLOT1), as well as EGFR, PI3K, Akt, and STAT3 (Zhang et al., 2023).

*Arctium lappa L.* is an edible medicinal plant. Arctigenin is a dibenzyl butyrolactone lignan which extracted from *Arctium lappa L.* (Wang et al., 2023). Previous studies investigating the potential anti-tumor effects of arctigenin using HCC cell lines, and luciferase assays have shown that arctigenin targeted the −450 to −400 region of the ankyrin promoter. This region is also a potential binding site for C/EBPα (a putative tumor suppressor that is upregulated in HCC subsets and required for growth and proliferation of cells). HepG2 and Hep-3B cells were treated with 20 μM arctigenin, and the co-immunoprecipitation assays have shown that the presence of arctigenin increases the binding between C/EBPα and PPAR α. Arctigenin can negatively regulate gankyrin by promoting the binding of C/EBPα and PPAR α, thereby inhibiting HCC (Sun et al., 2018). In addition, researchers have found that the ethanolic extract of *Arctium lappa L.* root also attenuates MASH-related hepatocarcinogenesis. The administration of *Arctium lappa L.* reduced the total FAs and lipid hydroperoxide levels in a steatohepatitis-induced HCC model (Romualdo et al., 2020).

Emodin is extracted from Chinese herbs, such as *Magnolia champaca* (L.) Baill*.* ex Pierre and *Reynoutria japonica* Houtt., which can affect the synthesis of FAs (Lee K. H. et al., 2017; Guo Y. et al., 2022). Bioinformatics analysis has shown that emodin effectively inhibits the growth and movement of HCC cells (Gao et al., 2024; Zhou et al., 2019). Emodin-treated human HCC cell, Bel-7402, has been used to study intrinsic lipid production. The researchers treated HCC cell line with different concentration of emodin (0, 25, 50, 100, 200, 400, and 600 μmol/L). After examining the cell viability, 100 μmol/L was considered the optimal concentration and experiments were performed. They found that the TG levels and FA desaturation in Bel-7402 cells decreased upon exposure to emodin. In addition, the expression levels of SREBP1 mRNA, and acid metabolism-related proteins (ACC α, FASN, and stearoyl-CoA A desaturase D) were also reduced. These findings indicate that emodin could promote apoptosis of HCC cells by regulating lipid metabolism in an SREBP1-dependent manner (Yang et al., 2019).

Curcumin (CUR), isolated from *Curcuma aromatica* Salisb., has an inhibitory effect on inflammation, oxidation, and tumors. It exhibits therapeutic benefits in various types of cancers (Bai et al., 2022; Shelash Al-Hawary et al., 2023). In a study based on network pharmacology of CUR-related mechanisms against HCC, scientists treated HepG2 cells with different concentrations of CUR (0, 20, 40, and 60 μmol/L), and the results revealed that CUR treated HCC by regulating the PI3K-Akt pathway, AMPK pathway, apoptosis and autophagy. It promoted HepG2 cell apoptosis through the p53 pathway and HepG2 autophagy through the AMPK/ULK1 pathway (Chen et al., 2022). Another study used CUR combining with sorafenib to treat H22 bearing mice and the results indicated that CUR prevented HCC progression by downregulating FASN and reducing lipid synthesis. The effect of CUR on lipid metabolism disorders is based on the PI3K/Akt pathway (Man et al., 2020).

Arenobufagin (ArBu), a bufadienolide isolated from *Ampelopsis delavayana* var. glabra (Diels & Gilg) C.L.Li, exhibits broad-spectrum anti-tumor activity (Chen K. et al., 2023). ArBu regulates lipid homeostasis in HCC cells. HepG2 cells and a xenograft model were used to assess the anti-HCC activity of ArBu. Scientists treated HepG2 cells with ArBu at 1.1 ng/mL and 3.3 ng/mL. The results showed that the effect was better at a concentration of 3.3 ng/mL. ArBu decreased the expression of 65 differentially expressed proteins related to autophagy, apoptosis, and lipid metabolism. This may be related to the arbus glycerophospholipid pathway, which is closely related to the PI3K/Akt pathway and JAK-STAT3 signaling pathway and is an important pathway for ArBu to affect lipid homeostasis (Zhao et al., 2020).

Berberine, an isoquinoline alkaloid extracted from *Coptis chinensis* Franch., promotes autophagic death in cancer cells (La et al., 2017). It has been reported that berberine can effectively inhibit HCC (Shou and Shaw, 2023). Scientists have isolated and synthesized several berberine analogues (A1-A13) and studied their roles in TG production in HepG2 cells. Berberine at a dose of 15 μM and berberine substituted by 9-O-benzoyl decreased intracellular TG content in HepG2 cells by activating AMPK, a major regulator of lipid metabolism (Cao et al., 2018).

## 6 Conclusion and perspectives

As a refractory malignant tumor that accounts for a large proportion of human cancers, HCC has been attracting much attention. Although the presence of abnormal lipid metabolism in HCC has been confirmed by numerous studies, there are still some contradictions in the results of HCC-related lipid levels, which make it difficult to further elucidate the relationship between HCC and lipid metabolism. The contradictions in the results may be related with the differences in testing sites, test method, evaluation and inclusion criteria, et al. In the future, more rigorous and standardized experimental design as well as testing and analysis techniques in the research need to be used. TCM formulae and active components has shown unique value on the journey against HCC. Some TCM formulae and active components have been proven in clinical and experimental studies to restore abnormal lipid metabolism in HCC, inhibit HCC progression and improve the life quality of the patients. However, research on the regulation of abnormal lipid metabolism in HCC by TCM formulae and active components is still in its nascent stages, with many aspects requiring in-depth exploration. Firstly, the majority of the current studies remain at the fundamental research stage, and the clinical evidence is obviously insufficient. More multicenter clinical trials are necessary to validate the efficacy and advantages of TCM formulae and their active components in restoring abnormal lipid metabolism in HCC. Secondly, current research on the regulation of HCC lipid metabolism by TCM formulae and active components is mostly limited to the formulae and active components themselves. It is also worth exploring whether their combination with existing drugs or biological agents can help better restore abnormal lipid metabolism in HCC. Recently, research in this field began to appear. For example, compound sophora injection is a formula approved by National Medical Products Administration in China, it could alleviate TAM-mediated immunosuppression with TNFR1 and sensitize HCC to sorafenib (Yang et al., 2020). Thirdly, whether the effect of a formula is accidental or not has become a question and needs to be proved by more research evidence. The quality standards, formulations, and usage methods of TCM formulae and active components also need to be further standardized to ensure the accuracy and reproducibility of experimental results. Fourthly, it is still unclear which specific substances in TCM formulae and active components play pivotal roles in regulating abnormal lipid metabolism in HCC, and their specific targets and pathways of action are also unknown. Emerging technologies such as multi-omics integration, high-throughput screening and computational modeling will help overcome this challenge. Besides, recent studies have reported the changes of lipid composition in exosomes from HCC patients, which will become the candidate biomarkers for early detection and treatment of HCC. Nevertheless, whether TCM formulae and active components can regulate the lipid composition in exosomes of HCC is still unknown, which will become a novel research field in the future. Overall, TCM formulae and active components have great promise in the treatment of HCC. In-depth research on the specific mechanism of them in the regulation of abnormal lipid metabolism in HCC will be helpful for the development of more effective therapeutic strategies for HCC.

## Glossary

AA: Arachidonic acid
ACC: ATP citrate lyase
ACLY: Acetyl-CoA carboxylase
AMPK: AMP-activated protein kinase
ApoA-1: Apolipoprotein A1
ArBu: Arenobufagin
BAs: Bile acids
CM: Chylomicrons
CPT: Carnitine palmitoyltransferase
CUR: Curcumin
CYP8B1: Cytochrome P450 family 8 subfamily B member 1
DMY: Dihydromyricetin
EMT: Epithelial-mesenchymal transition
FAs: Fatty acids
FXR: Farnesoid X receptor
FZXZP: Fuzheng Xiaozheng prescription
FZYLD: Fuzheng Yiliu Decoction
GFL: Ganfule capsules
HBV: Hepatitis B virus
HCC: Hepatocellular carcinoma
HCV: Hepatitis C virus
HDL: High-density lipoproteins
HDLBP: High-density lipoprotein-binding protein
HMG-CoA: 3-hydroxy-3-methyl-glutaryl-coenzyme A
IDL: Intermediate-density lipoprotein
LDL: Low-density lipoproteins
LDL-C: Low-density lipoprotein cholesterol
Lp (a): Lipoprotein (a)
MASH: Metabolic dysfunction-associated steatohepatitis
MASLD: Metabolic dysfunction-associated steatotic liver disease
MUFAs: Monounsaturated fatty acids
NKT: Natural killer T
PPAR: Peroxisome proliferator-activated receptor
PUFAs: Polyunsaturated fatty acids
PZH: Pien Tze Huang
ROS: Reactive oxygen species
RXRB: Retinoid X receptor β
SCD: Stearyl-CoA desaturase
SFAs: Saturated fatty acids
SHHZF: Shuihonghuazi Formula
SNKAF: Sinikangai fang
SREBP1: Sterol regulatory element-binding protein 1
TAMs: Tumor-associated macrophages
TCM: Traditional Chinese medicine
TG: Triglycerides
TME: Tumor microenvironment
UFAs: Unsaturated fatty acids
VLC: Very long chain
VLDL: Very-low-density lipoproteins
